# A Functional Monomer Is Not Enough: Principal Component Analysis of the Influence of Template Complexation in Pre-Polymerization Mixtures on Imprinted Polymer Recognition and Morphology

**DOI:** 10.3390/ijms151120572

**Published:** 2014-11-10

**Authors:** Kerstin Golker, Björn C. G. Karlsson, Annika M. Rosengren, Ian A. Nicholls

**Affiliations:** 1 Bioorganic & Biophysical Chemistry Laboratory, Linnaeus University Centre for Biomaterials Chemistry, Linnaeus University, SE-391 82 Kalmar, Sweden; E-Mails: kerstin.golker@lnu.se (K.G.); bjorn.karlsson@lnu.se (B.C.G.K.); annika.rosengren@lnu.se (A.M.R.); 2 Department of Chemistry—BMC, Uppsala University, SE-751 23 Uppsala, Sweden

**Keywords:** molecularly imprinted polymer, molecular dynamics, molecular recognition, molecular imprinting, principal component analysis, chemometrics

## Abstract

In this report, principal component analysis (PCA) has been used to explore the influence of template complexation in the pre-polymerization phase on template molecularly imprinted polymer (MIP) recognition and polymer morphology. A series of 16 bupivacaine MIPs were studied. The ethylene glycol dimethacrylate (EGDMA)-crosslinked polymers had either methacrylic acid (MAA) or methyl methacrylate (MMA) as the functional monomer, and the stoichiometry between template, functional monomer and crosslinker was varied. The polymers were characterized using radioligand equilibrium binding experiments, gas sorption measurements, swelling studies and data extracted from molecular dynamics (MD) simulations of all-component pre-polymerization mixtures. The molar fraction of the functional monomer in the MAA-polymers contributed to describing both the binding, surface area and pore volume. Interestingly, weak positive correlations between the swelling behavior and the rebinding characteristics of the MAA-MIPs were exposed. Polymers prepared with MMA as a functional monomer and a polymer prepared with only EGDMA were found to share the same characteristics, such as poor rebinding capacities, as well as similar surface area and pore volume, independent of the molar fraction MMA used in synthesis. The use of PCA for interpreting relationships between MD-derived descriptions of events in the pre-polymerization mixture, recognition properties and morphologies of the corresponding polymers illustrates the potential of PCA as a tool for better understanding these complex materials and for their rational design.

## 1. Introduction

Molecular imprinting is a technique widely used for preparing ligand-specific polymeric materials with antibody-like recognition properties [1,2,3,4,5]. Since molecularly imprinted polymers (MIPs) are robust materials [6] displaying ligand selectivities, sometimes challenging their biological counterparts, interest in employing these materials in applications, such as biomimetic assays- and sensors [7,8,9] and as solid phase extraction agents [10,11,12] is rapidly increasing.

The nature and extent of the molecular interactions present in the pre-polymerization phase provide the basis for the recognition properties of MIPs and have been investigated in detail using a variety of spectroscopic [13,14,15,16,17] and theoretical [18,19,20,21,22,23] approaches. More recently, the use of molecular dynamics (MD) simulations for investigating the molecular level events in MIP pre-polymerization mixtures [22,24,25,26,27,28,29] has attracted increasing interest. Such studies employ multiple explicit replicates of all molecular species present in the pre-polymerization mixture with the same stoichiometries used in synthesis protocols [25,26,27,28,29].

However, the relationships between the factors influencing the recognition qualities of MIPs are difficult to study, since evaluation with conventional univariate methods is limited. This limitation can be overcome by the use of multivariate chemometric methods, in which mathematical and statistical methods are applied to chemical data. The methods allow for the simultaneous assessment of several factors, as opposed to the more common single variable studies of univariate methods. One multivariate method is principal component analysis (PCA), a data analysis technique for the reduction of multi-dimensional datasets to lower dimensions [30]. A PCA can be used to discover patterns in complex data matrices and can reveal relationships between measurements and variables, as well as between the variables themselves, which otherwise are likely to remain undetected [31].

Despite the advantages of using multivariate methods when optimizing polymer composition and template-MIP binding parameters, only a few efforts have been reported in the literature [32,33,34,35,36,37,38,39,40,41,42,43,44,45,46,47,48]. Moreover, to the best of our knowledge, no attempts have been made to apply chemometrics on MIP pre-polymerization data obtained from MD simulation trajectories.

We have recently reported on the influence of polymer composition on morphology and template recognition [27]. The polymer systems used in the present study were copolymers of the crosslinking agent ethylene glycol dimethacrylate (EGDMA) prepared with either methacrylic acid (MAA) or methyl methacrylate (MMA) as the functional monomer and imprinted with the local anesthetic, bupivacaine. In total, 16 MIP and corresponding non-imprinted reference polymer (REF) systems were included in the analysis. The polymers were prepared with different molar ratios of the respective monomers, though the stoichiometries of the MAA and MMA polymer series were equivalent (Table 1 and Table 2). The choice of MMA as a functional monomer was based upon an interest in observing the impact of removing the capacity of the functional monomer to donate hydrogen bonds. PCA was applied to data extracted from MD simulations of the respective all-component pre-polymerization mixtures, morphology characterization (surface area and porosity) and recognition behavior of the corresponding bulk polymers.

**Table 1:** Compositions of the molecularly imprinted polymers prepared with methacrylic acid (MAA) (in mmol) ^a^. MIP, molecularly-imprinted polymer; EGDMA, ethylene glycol dimethacrylate; AIBN, 2,2'-azobis-(2-methylpropionitrile).

Polymer	Bupivacaine	MAA	EGDMA	AIBN	Toluene	Molar Ratio ^b^
MIP 0	1.39	0.00	77.4	2.00	219.8	1:0:56
MIP 1	1.39	3.30	78.1	2.10	226.0	1:2:56
MIP 2	1.39	7.23	74.5	2.03	221.5	1:5:54
MIP 3	1.39	12.51	77.4	2.18	236.4	1:9:56
MIP 4	1.39	16.68	77.4	2.23	240.9	1:12:56
MIP 5	1.39	20.1	82.3	2.40	259.4	1:14:59
MIP 6	1.39	22.24	77.4	2.30	246.9	1:16:56
MIP 7	1.39	25.02	77.4	2.30	251.4	1:18:56
MIP 8	1.39	30.90	82.6	2.55	274.0	1:22:59
MIP 9	1.39	64.20	76.2	2.80	298.4	1:46:55

^a^ Non-imprinted reference polymers were also prepared, though in the absence of bupivacaine. ^b^ Bupivacaine:MAA:EGDMA.

**Table 2:** Composition of the molecularly imprinted polymers prepared with methyl methacrylate (MMA) (in mmol) ^a^.

Polymer	Bupivacaine	MMA	EGDMA	AIBN	Toluene	Molar Ratio ^b^
MIP 0	1.39	0.00	77.4	2.00	219.8	1:0:56
MIP 10	1.39	7.23	77.4	2.18	236.4	1:7:56
MIP 11	1.39	13.14	77.4	2.23	240.9	1:9.5:56
MIP 12	1.39	16.90	77.4	2.30	246.9	1:12:56
MIP 13	1.39	19.72	77.4	2.30	251.4	1:14:56
MIP 14	1.39	30.86	77.4	2.40	269.5	1:22:56
MIP 15	1.39	64.22	77.4	2.80	322.2	1:46:56

^a^ Non-imprinted reference polymers were also prepared, though in the absence of bupivacaine. ^b^ Bupivacaine:MMA:EGDMA.

The analysis revealed that the molar fraction functional monomer in the MAA-polymers contributed to describing both the binding and morphology features. Moreover, competition in the hydrogen bond contact to the template between the functional monomer and the crosslinker in the pre-polymerization phase was revealed in both polymer series. Interestingly, weak correlations between the swelling behavior and the rebinding characteristics of the MAA-MIPs were observed. Furthermore, the results highlight the potential of using MMA as a non-crosslinking analogue of EGDMA for future studies on the impact of the degree of cross-linking on the behavior of EGDMA-based polymers. 

## 2. Results and Discussion

In this study, the influence of template-monomer complexation in the pre-polymerization phase on polymer-template recognition and morphology was investigated through the use of PCA. The polymer systems studied were bupivacaine-imprinted EGDMA-copolymers containing different amounts of either MAA or MMA as the functional monomer. The analyzed data were obtained from equilibrium binding experiments (Tables S1 and S2), gas sorption measurements (Tables S3 and S4), swelling studies (Tables S5 and S6) and from MD simulation trajectories of all-component pre-polymerization mixtures with molar ratios corresponding to the synthesized bulk polymers (Tables S7–S12). 

The variables analyzed were the molar fraction MAA (Mfrac_MAA_) and MMA (Mfrac_MMA_) employed in the polymer systems, descriptors of the rebinding behavior expressed as the fraction of radioligand bound to the MIP (B/T_MIP_) and to the REF (B/T_REF_), as well as the specific binding (B/T_Spec_), which is defined as the difference between B/T_MIP_ and B/T_REF_. Additional variables employed in the analysis were the BET surface area of the MIPs (BSA_MIP_) and the REFs (BSA_REF_), the Langmuir surface area of the MIPs (LSA_MIP_) and the REFs (LSA_REF_), the pore volume of the MIPs (PV_MIP_) and the REFs (PV_REF_), as well as the swelling ratio of the MIPs (SW_MIP_) and the REFs (SW_REF_).

The variables describing the data extracted from MD simulation trajectories were measures of the hydrogen bond contact between the molecules present expressed as the percentage of the total simulation time (Tables S11 and S12): the average hydrogen bond contact between bupivacaine and the respective functional monomer MAA or MMA (%Bup-funcM), the average hydrogen bond contact between bupivacaine and the crosslinker EGDMA (%Bup-EGDMA), bupivacaine self-association (%Bup-Bup), contact between the amide proton functionality (HAB) of bupivacaine and the carbonyl oxygens (OAD) of either MMA or MAA (%HAB-OAD), as well as the contact between the amide proton of bupivacaine and a carbonyl oxygen (OAF) of EGDMA (%HAB-OAF). Both the total average hydrogen bond contact between the different molecules and the contact between the specific interaction points on the individual molecules were employed as variables in the PCA, since the total average hydrogen bond contact is the sum of the specific interactions between the individual molecules. From Tables S11 and S12, it can be seen that the hydrogen bond contact between the specific interaction points on the individual molecules contributes differently to the total average contact between the molecule species, in particular to the total average contact between the template bupivacaine and the two different functional monomers, MAA and MMA. In a previous report, it was suggested that the strength of the hydrogen bond contact between the amide proton functionality of bupivacaine and the carbonyl oxygen of MAA was steering both binding capacity and the specific binding in a series of MAA-polymers [27]. See Figure 2 for atom designations.

**Figure 1:**
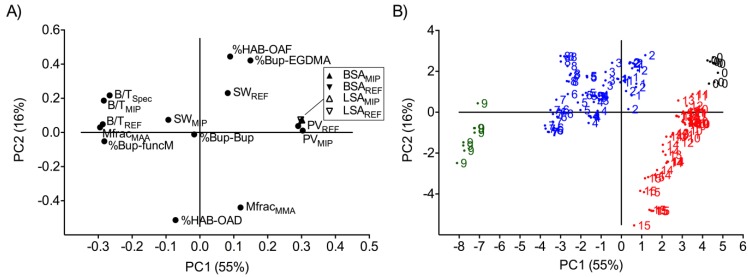
(**A**) Loading plot of data from equilibrium rebinding studies, swelling studies, morphology characterization and MD (molecular dynamics) simulations on the methacrylic acid (MMA) and methyl methacrylate (MAA) polymer systems; (**B**) score plot of data showing grouping with respect to the functional monomer used in the respective polymer system. Blue and green: Polymers 1–9 prepared with MAA; red: Polymers 10–15 prepared with MMA; black: Polymer 0 prepared without the functional monomer. See Table 1 and Table 2 for the composition of the polymers. Molar fraction MAA, Mfrac_MAA_; molar fraction MMA, Mfrac_MMA_; fraction radioligand bound to MIPs, B/T_MIP_; fraction radioligand bound to REFs, B/T_REF_; specific binding, B/T_Spec_; BET surface area of MIPs, BSA_MIP_; BET surface area of REFs, BSA_REF_; Langmuir surface area of MIPs, LSA_MIP_; Langmuir surface area of REFs, LSA_REF_; pore volume of MIPs, PV_MIP_; pore volume of REFs, PV_REF_; swelling ratio of MIPs, SW_MIP_; swelling ratio of REFs, SW_REF_; average hydrogen bond contact between bupivacaine and functional monomer (MAA or MMA), %Bup-funcM; average hydrogen bond contact between bupivacaine and EGDMA, %Bup-EGDMA; bupivacaine self-association, %Bup-Bup; contact between amide proton functionality (HAB) of bupivacaine and carbonyl oxygens (OAD) of either MMA or MAA, %HAB-OAD; contact between amide proton of bupivacaine and carbonyl oxygen (OAF) of EGDMA, %HAB-OAF. See Figure 2 for atom labels.

In PCA, multivariate data are projected onto a low-dimensional plane, where, e.g., groups of observations and trends can be revealed. The different principal components (PCs) are vectors in the n-dimensional variable space computed in such a way as to best approximate the data in the least squares sense. The first principal component (PC1) passes through the average point (the origin) in the data space pointing in the direction of maximum variance in the variable dataset. The second PC (PC2) is orthogonal to PC1, passes also through the origin and points in the direction of the second maximum variance in the dataset. A plot of the residual variance is shown in Figure S1. The two PCs define a two-dimensional plane onto which each data point is projected with a new coordinate value. The projections of the observed data (measured values of a sample) are called scores, and the projections of the variables are called loadings. Accordingly, the loading plot (Figure 1A) reveals correlations between the variables, and the score plot (Figure 1B) describes correlations between the observed data. Variables with high loadings (far from the origin) describe most of the variation in the data. The variables that are located closely together are highly correlated [30,31]. If they share the same sign, the correlation is positive, and if they have opposite signs, the correlation is negative. The score plot describes the measured properties of the samples (polymers), where grouping of the scores indicates that the samples share the same characteristics [30,31].

The morphological characteristics of the polymers (surface area and pore volume) together with the binding properties, the molar fraction of MAA and the hydrogen bond contact between functional monomer and bupivacaine in the pre-polymerization phase account for most of the variation in the data underlying PC1 (55%). The evident separation of the loadings corresponding to the binding properties and those corresponding to the morphological features in PC1 demonstrates the importance of taking morphological aspects into account when characterizing the binding performance of MIPs.

**Figure 2:**
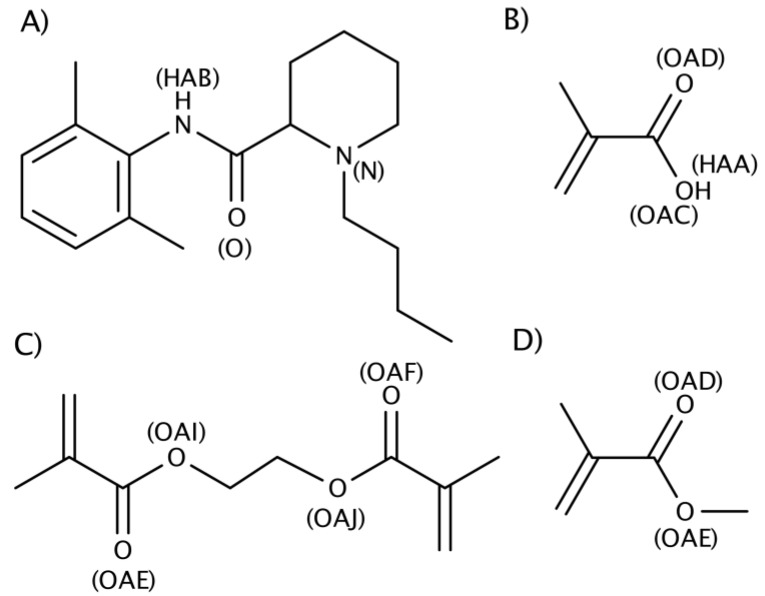
The structures of compounds used in the study and the designations of atoms potentially participating in hydrogen bond interactions: (**A**) Bupivacaine; (**B**) MAA; (**C**) EGDMA; (**D**) MMA.

The loading describing the averaged bupivacaine-functional monomer hydrogen bond interactions (%Bup-funcM) is located close to that describing the molar fraction MAA in the polymers (Mfrac_MAA_), suggesting strong positive correlation between these factors. This can be explained by the fact that the averaged hydrogen bond contact between MAA and bupivacaine is higher than that between MMA and bupivacaine due to the additional interaction points provided by the acidic proton at MAA’s carboxyl functionality (Figure 2). Further, it can be seen from the loading plot (Figure 1A) that the molar fraction MAA in the polymers is positively correlated to the binding of bupivacaine to both the MIPs and the REFs in accordance with the results presented previously [27], implying higher binding with increased molar fraction MAA. The molar fraction MMA, in contrast, is negatively correlated to the variables describing binding and positively correlated to surface area and pore volume. However, the loading of Mfrac_MMA_ on PC1 is lower compared to the loading of Mfrac_MAA_, indicating that the molar fraction MMA in the polymers does not contribute as much as the molar fraction MAA to the variation in the dataset. It is worth noting that the descriptors of the surface area for both the MIPs and the REFs derived with the BET and the Langmuir methods contribute equally to the variation in the data. Moreover, weak positive correlations between the swelling behavior and the rebinding characteristics of the MAA-MIPs were revealed, as the corresponding variables are located in the same quadrant of the loading plot.

PC2 denotes 16% of the variation in the data that can predominantly be ascribed to the interactions between functional and crosslinking monomers and the template in the pre-polymerization phase. These interactions arise from hydrogen bond contacts between the carbonyl oxygens of MAA, MMA or EGDMA and the amide proton of bupivacaine (Figure 1, Tables S11 and S12). The results clearly demonstrate the presence of competition for hydrogen bonding to the template between the carbonyl interaction points of the respective functional monomer, MAA or MMA, and EGDMA, since the descriptors of these interactions are positioned in diagonally located quadrants of the plot, thus being negatively correlated. This provides a unique illustration of the diversity of the interactions between pre-polymerization mixture components and the template. Interestingly, the binding of the template to the MIPs (B/T_MIP_) and the specific binding (B/T_Spec_) were separated in PC2 from the binding to the non-imprinted reference polymers (B/T_REF_).

In the score plot, three groupings could be identified (Figure 1B). The groupings of the scores are related to which functional monomer was used during synthesis and also to its molar fraction. Interestingly, the scores corresponding to the MMA polymers (Polymers 10–15, red scores) and those corresponding to the polymer not containing any functional monomer (Polymer 0, black scores) can be considered as one group, which demonstrates that the MMA polymers and the polymer prepared without the functional monomer share similar properties. These polymers are correlated to the descriptors of morphology where the spread of their scores in PC1 is of minor magnitude. In contrast, the spread of the scores corresponding to these polymers in PC2 is more pronounced. This can be ascribed to the increasing molar fraction MMA in the polymers. These results indicate that the increase of the molar fraction MMA in the polymers affects the morphological features (surface area and pore volume) only to a minor extent. Further, the similarity of the properties of the MMA polymers and the polymer prepared without a functional monomer suggests that MMA may be considered as a non-crosslinking analogue of EGDMA, of interest for studies of the influence of the degree of crosslinking in these polymers, a topic of on-going work.

In the case of the MAA polymers (Figure 1B, blue and green scores), the scores were spread in PC1 according to the increasing molar fraction MAA in the polymers, the positively correlated rebinding behavior to both the MIPs and the REFs and the positively correlated hydrogen bond contact between template and functional monomer. Polymer 9, containing the highest molar fraction MAA within the studied polymer series, displayed the highest score values in PC1. This polymer has previously been shown to display the highest binding capacity and the most frequent hydrogen bond contact between template and functional monomer in the pre-polymerization phase [27]. The distribution of the scores in relation to the location of the loadings suggests that binding to both the MIPs and the REFs, as well as the specific binding, increases with increasing molar fraction MAA in the polymers, in accordance with results presented previously [27].

In summary, the polymers containing MMA as a functional monomer and the one prepared without functional monomer share the same properties. The rebinding capacity of these polymers can be interpreted as equally low, and the morphological characteristics, such as the surface area and pore volume, were comparable and rather independent from the molar fraction MMA used in the synthesis. The molar fraction of functional monomer in the MAA-polymers, in contrast, contributed to describing both binding and morphology features. Interestingly, the analysis exposed competition in the hydrogen bond contact to the template between the functional monomer and the crosslinker in both the simulated MMA- and MAA pre-polymerization mixtures.

## 3. Experimental Section 

### 3.1. Chemicals

[^3^H]-(*R*,*S*)-bupivacaine (specific activity 2.7 Ci/mmol) was obtained from Moravek Biochemicals Inc. (Brea, CA, USA). (*R*,*S*)-bupivacaine hydrochloride, methyl methacrylate (MMA) and methacrylic acid (MAA) were purchased from Sigma-Aldrich (Steinheim, Germany) and toluene from Merck (Solna, Sweden). Ethylene glycol dimethacrylate (EGDMA) was obtained from Fluka (Buchs, Switzerland) and 2,2'-azobis-(2-methylpropionitrile) (AIBN) from Janssen Chimica (Geel, Belgium). All chemicals were of analytical grade, and the water used was of Millipore quality (Millipore AB, Solna, Sweden).

### 3.2. Data Analysis

The PCA was performed using the software package The Unscrambler v.10.2 (Camo Software AS, Oslo, Norway). A total of 2,964 data points was used in the study. For the binding study 9 replicates were performed; for the morphology experiments 1 batch of bulk polymer was used for each composition; and the MD data were extracted from molecular trajectories obtained from five simulations on each system. Prior to data analysis, the variables were mean centered and then scaled using the standard deviation in order to provide equal weighting to the variables.

### 3.3. Molecular Dynamics (MD) Simulations

All-atom MD simulations of the polymer systems were performed as described previously [25,26,27] using the AMBER (v.10.0 UCSF, San Francisco, CA, USA) platform of programs [49,50]. The simulated pre-polymerization mixtures differed in composition through variations of the molar ratio of the monomers employed. The molar fraction functional monomer (either MMA or MAA) was increased over the range of simulated molar ratios, while the other components where held effectively constant. For detailed information regarding the compositions of the simulated all-component pre-polymerization mixtures, as well as for equilibration and production run data, see Tables S7–S10. All systems were simulated in quintuplicate, each covering 10 ns of recorded trajectory data for each mixture (totally 50 ns). Final equilibrated trajectories were analyzed using the PTRAJ module implemented in AmberTools (v.1.3 UCSF, San Francisco, CA, USA). Hydrogen bond interactions were extracted from the trajectories using a cut-off distance and angle of 3.0 Å and 120°, respectively. The structures of the molecular species and the designations of atoms potentially participating in hydrogen bond interactions are presented in Figure 2.

For the average hydrogen bond occupancies for all analyzed atom-pairs in each system and the corresponding averaged lifetimes, see Tables S11 and S12.

### 3.4. Polymer Synthesis

A series of bupivacaine-imprinted polymers containing either MMA or MAA as the functional monomer were synthesized and treated as described previously [27,51]. Corresponding non-imprinted reference (REF) polymers were prepared following the same procedure. The compositions of the polymers (Table 1 and Table 2) and their stoichiometries were equivalent to those of the pre-polymerization mixtures simulated by MD (Tables S7 and S8). 

### 3.5. Equilibrium Binding Study

Binding to both the MIP and the REF polymers was studied at a polymer concentration of 0.75 mg/mL. The radioligand, [^3^H]-(*R,S*)-bupivacaine (15 pmol/mL), and polymer suspended in toluene were mixed in a total volume of 1 mL. The samples were incubated on a rocking table at 293 K for 3 h. Thereafter, they were centrifuged (7,000× *g* for 5 min), and the supernatant (600 μL) was mixed with the scintillation cocktail (2 mL, Beckman Ready Safe, Beckman Caulter, Bromma, Sweden). The activity was then measured for 2 min by scintillation counting (Packard Tri-Cab 2100TR liquid scintillation counter, PerkinElmer, Zaventem, Belgium). Control samples containing no polymer were also prepared and treated identically. All samples were analyzed in triplicate (Tables S1 and S2).

### 3.6. Swelling Studies

A volume of 1 mL dry polymer (*V_0_*) was measured in a glass cylinder, and after the addition of an excess of toluene, the cylinder was sealed. The volume of the swollen polymer (*V_SW_*) was recorded after 24 h. The swelling ratio (*SW*) was calculated according to Equation (1) (Tables S5 and S6).

(1) $$SW=\;\frac{V_{SW}\;-\;V_{0}}{V_{0}}\;\times \;100$$

### 3.7. Examination of Gas Accessible Surface Areas and Porosities

Polymer surface area and porosity were examined with nitrogen sorption studies by the Brunauer, Emmet and Teller [52] (BET), the Langmuir [53] and the Barrett, Joyner and Halenda [54] (BJH) methods (Tables S3 and S4). Prior to the measurements, samples were degassed at 323 K for 24 h to remove adsorbed gases and moisture. BET-and Langmuir surface areas were calculated from the adsorption data using 0.162 nm^2^ as the molecular cross-sectional area for adsorbed nitrogen molecules. The BJH method was applied to calculate the pore volumes of the polymers from the desorption branch of the isotherms. All measurements were performed on an ASAP 2004 instrument (Micromeritics, Norcross, GA, USA) at 77 K.

## 4. Conclusions

In this study, a PCA was performed on data extracted from all-component MD simulation trajectories of a series of molecularly imprinted pre-polymerization mixtures together with data describing the surface characteristics and rebinding behaviors of analogous synthesized bulk polymers. Unique insights concerning the influence of template-monomer complexation on recognition were gained.

The polymers prepared with MMA as a functional monomer were, through the analysis, revealed to share the same properties as the polymer prepared without any functional monomer, which highlights the potential of the use of MMA as a non-crosslinking analogue of EGDMA. The rebinding capacities of these polymers were equally low, and the morphological characteristics, such as surface area and pore volume comparable and rather independent of the molar fraction MMA used in synthesis. The molar fraction functional monomer in the MAA-polymers, on the other hand, contributed to describing both the binding, surface area and pore volume. Interestingly, weak positive correlations between the swelling behavior and the rebinding characteristics of the MAA-MIPs were revealed. 

Finally, the results presented in this report demonstrate the potential of multivariate methods for analyzing the characteristics of complex materials, e.g., MIPs, where unique insights of importance for the further development of these materials may be gained

## Supplementary Materials

Supplementary materials can be accessed at: http://www.mdpi.com/1422-0067/15/11/20572/s1.
